# Nitrogen Starvation Differentially Influences Transcriptional and Uptake Rate Profiles in Roots of Two Maize Inbred Lines with Different NUE

**DOI:** 10.3390/ijms20194856

**Published:** 2019-09-30

**Authors:** Maria Mascia, Davide Sega, Anita Zamboni, Zeno Varanini

**Affiliations:** Biotechnology Department, University of Verona, Strada le Grazie 15, 37134 Verona, Italy; mariagraziamascia@hotmail.it (M.M.); davide.sega@univr.it (D.S.); zeno.varanini@univr.it (Z.V.)

**Keywords:** maize, N shortage, microarray

## Abstract

Nitrogen use efficiency (NUE) of crops is estimated to be less than 50%, with a strong impact on environment and economy. Genotype-dependent ability to cope with N shortage has been only partially explored in maize and, in this context, the comparison of molecular responses of lines with different NUE is of particular interest in order to dissect the key elements underlying NUE. Changes in root transcriptome and NH_4_^+^/NO_3_^−^ uptake rates during growth (after 1 and 4 days) without N were studied in high (Lo5) and low (T250) NUE maize inbred lines. Results suggests that only a small set of transcripts were commonly modulated in both lines in response to N starvation. However, in both lines, transcripts linked to anthocyanin biosynthesis and lateral root formation were positively affected. On the contrary, those involved in root elongation were downregulated. The main differences between the two lines reside in the ability to modulate the transcripts involved in the transport, distribution and assimilation of mineral nutrients. With regard to N mineral forms, only the Lo5 line responded to N starvation by increasing the NH_4_^+^ fluxes as supported by the upregulation of a transcript putatively involved in its transport.

## 1. Introduction

Nitrogen (N) is the mineral nutrient required by plants in the largest amount for growth and development. In fact, it represents 1–5% of total plant dry matter as an integral constituent of proteins, nucleic acids, chlorophyll, co-enzymes, phytohormones and secondary metabolites [1]. Crop production heavily depends on N fertilization that, in the last 50 years, has been the most efficient method to increase yield [2]. However, due to the low nitrogen use efficiency (NUE) of the crops—less than 50% as estimated by Baligar et al. [3]—this has a negative impact from economic and environmental points of view.

Nitrogen use efficiency (NUE) is a complex trait constituted by two main components, N uptake (NUpE) and N utilization efficiency (NUtE), and involves biochemistry, phenology, architecture and responses to the environment [4,5]. It was reported that the role of NUpE and NUtE in determining the overall NUE is influenced by the N supply condition [6]. An emergent exigence is the improvement of NUpE due to its low value in general observed for cereals [6]. Crops usually acquire N from the soil mainly through the two inorganic forms, nitrate (NO_3_^−^) and ammonium (NH_4_^+^). Mechanisms involved in root uptake of these forms can have a pivotal role in NUpE. In rice, NUE differences were observed in relation to the polymorphism of a *NRT1.1B* gene encoding a NO_3_^−^ transporter that is also involved in the signaling of the same anion [7]. Besides, an increase of yield was observed in the same species in response to the overexpression of *OsNRT2.3,* a gene encoding an high-affinity NO_3_^−^ transporter, although it has been shown that the effect was more correlated to the pH sensing rather than the transport of the anion [8]. Concerning maize, a study of the impact of different supply of N on NO_3_^−^ and NH_4_^+^ uptake systems was carried out in 27 genotypes showing the correlation of the variations in expression of the *ZmNRT2.2* (encoding a NO_3_^−^ high-affinity transporter) between limited and adequate N levels and the genotype-specific ability to maintain biomass under low N [6]. The same authors hypothesized a strong role of the high affinity NO_3_^−^ transporter (NRT2s) in enhancing NUpE [6].

Notwithstanding the importance of genes involved in N transport, other root mechanisms playing a role in the tolerance and ability to grow under N paucity could be linked to NUE. The analysis of molecular plant responses to low N condition or shortage can offer the possibility to identify key components at the basis of the capability to cope with N limited availability conditions. Genome-wide transcriptional analyses offer the possibility to draw a picture of the main molecular processes involved in the responses to this nutritional condition. Several examples of this approach applied in root and shoot tissues were reported for Arabidopsis [9], poplar [10], rice [11], cucumber [12], maize [13] and wheat [14]. Concerning the comparison of transcriptional responses in different maize genotypes, a characterization in leaf expression profile changes under low N of two elite Chinese inbred lines’ growth in field under normal and low N conditions was provided [13]. In the present work we focused on the variations in root transcriptome of two maize inbred lines characterized by different NUE [15,16] during the growth in hydroponics in the absence of N. These lines, Lo5 (high NUE) and T250 (low NUE), were previously studied with regard to changes in their root transcriptome during NO_3_^−^ induction [17]. In this context, the two lines differed not only in the timing of the response to the treatment with the anion but also in the molecular strategies used in order to cope with changes of NO_3_^−^ availability in the environment [17]. Only in the low-NUE T250 that responded slower to induction in terms of uptake rate was a modulation of transcripts involved in the anion uptake and assimilation observed [17]. On the basis of these results, in the present work we studied the behavior of these two maize lines during growth without N with the aim to identify genotype-specific mechanisms playing a role in the response to N shortage.

## 2. Results and Discussion

The identification of biomarkers [18,19] and the analysis of gene regulatory networks [20] linked to N status in maize has been mainly restricted to leaf tissues. To dissect the root molecular responses to N starvation of the two maize inbred lines, Lo5 and T250, showing different NUE in field [15,16], we carried out a series of comparison of root genome-wide transcriptional profiles obtained through microarray analysis. In particular, we compared the root transcriptional profiles in both genotypes at 1 and 4 days (d) of hydroponic growth without N with that of seedlings sampled at 0 d (starting point of N deprivation). The differentially expressed transcripts identified for each comparison through t-test analysis are reported in Table 1 (*p*-value ≤ 0.01 and |Log_2_(ratio)|≥ 1; Table S1). The low NUE line, T250, differentially expressed twice transcripts than Lo5 after 1 d of N starvation. After 4 d, the Lo5 high NUE genotype modulated a slightly higher number of transcripts (591 vs. 482) in comparison to T250. Regarding the genotype-dependent transcriptome responses linked to N nutrition, it was reported a description of changes due to low N condition (0.2 mM vs. 2 mM) in two Tibetan wild barley genotypes exhibiting different tolerance to this nutritional stress [21]. When contrasting normal and low N conditions, twice as many genes were modulated in the tolerant barley line comparing to the susceptible one both at 6 and 48 h of treatment. Differences among these data and those observed in the present work could be ascribed to plant species and/or experimental conditions. Furthermore, Lo5 and T250 lines showed a different transcriptional behavior in terms of the number of modulated transcripts also in response to NO_3_^−^ treatment [17]. The differential expression of a selected set of transcripts identified through the four comparisons was confirmed by Real-time RT-PCR experiments (Table S2). Expression data of eight transcripts obtained with both techniques (microarray and Real-time RT-PCR) were congruent as suggested by the results of correlation analysis (R^2^ = 0.998, Figure S1). The selection of these transcripts was made considering their involvement in N (NO_3_^−^ and NH_4_^+^) uptake and other metabolic processes linked to nutrient homeostasis.

### 2.1. Maize Root Responses to Growth without N: Transcripts Commonly Modulated in Both Inbred Lines

**Figure 1:**
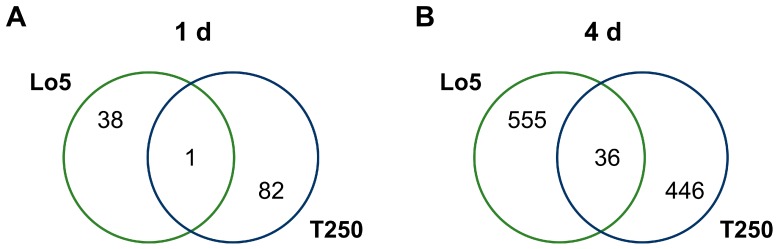
Shared and specific transcripts modulated during the growth without N. (**A**) Shared and specific transcripts modulated between Lo5 and T250 at 1 d. (**B**) Shared and specific transcripts modulated between Lo5 and T250 at 4 d.

Only a small number of transcripts (one at 1 d and 36 at 4 d) were commonly, and in the same direction, modulated by the two inbred lines during the growth without N (Figure 1A,B and Table 2). These results suggest that the two maize lines adopt different molecular strategies in root in order to respond to the shortage of N. Anyway, these commonly modulated transcripts represent the genotype-independent responses to this nutritional stress. Focusing on 4 d, we interestingly observed an upregulation of a transcript (*GRMZM2G162739_T01*) encoding a protein belonging to plant protein family NAC (no apical meristem (NAM), *Arabidopsis* transcription activation factor (ATAF), Cup-shaped cotyledon (CUC)) (Table 2) known to play a role in the control of responses to several biotic and abiotic stress [22]. Therefore, this transcription factor could be involved in the regulation of N shortage responses in the root of both lines. It has already been reported for poplar that the root responses to low N condition are linked to hierarchically structured genetic sub-networks and one of them is centered on *PtaNAC1* [10]. Furthermore, the modulation of this genes is linked to changes in root biomass and expressions of other connected hub genes under low N [10]. Indeed, other regulatory pathways can be involved in the changes induced in roots during the responses to N deficiency as suggested by the differential expression of genes encoding for basic-helix-loop-helix (bHLH), ETHYLENE RESPONSIVE ELEMENT BINDING FACTOR (ERF) and MYB transcription factors. We observed a downregulation of a transcript (*GRMZM2G120021_T01*; Table 2) for a bHLH homologous to an Arabidopsis protein (37% of amino acid identity; AT4G37850.1) encoded by a jasmonate (JA) responsive gene [23]. The expression of the JA responsive genes also including *JAZ* (jasmonate ZIM-domain) genes is controlled by JASMONATE-INSENSITIVE 3 (JAI3) and other JAZ proteins among which JAZ1 in Arabidopsis [23]. AtJAI3 and AtJAZ, whose levels are controlled by protein degradation induced by JA, negatively regulate the key transcriptional activator of jasmonate responses, AtMYC2. AtMYC2 activates JA responsive genes including JAZ genes as *JZ1*. Interestingly, we observed in both maize lines grown without N a positive modulation of a transcript (*GRMZM2G145458_T01*; Table 2) encoding a JAZ protein. On the basis of these results, it could be hypothesized that in both lines the response to N starvation is regulated by JA through a mechanism similar to that depicted for *Arabidopsis thaliana* that involves the activity of JAZ proteins and MYC2 in a feed-back loop explaining the rapid responses to JA and the subsequent rapid switch off [23]. Moreover, also ethylene can play a role in controlling maize root responses to growth without N as suggested by the upregulation of an ETHYLENE RESPONSIVE ELEMENT BINDING FACTOR (ERF) (*GRMZM2G080516_T01*; Table 2). In cucumber leaves, genes involved in JA and ethylene signal (e.g., ERFs) were reported to be positively affected between 3 and 6 h of N starvation [12]. Furthermore, it was hypothesized that ethylene and auxin signal could regulate the anthocyanin synthesis controlling the expression of a cucumber hub gene orthologous to *AtMYB12* [12]. Another MYB gene, *AtMYB112*, controls anthocyanins biosynthesis in *Arabidopsis* seedlings under salinity and high light stress but not in response to 6 d-N shortage [24]. In both inbred lines we recorded after 4 d of growth without N an overexpression of a transcript encoding a MYB protein (*GRMZM2G134279_T01*, Table 2) orthologous to *AtMYB112* (80% amino acid identity). Besides, two UDP-glucosyl transferase (UGT) transcripts were positively modulated (*GRMZM2G178209_T01* and *AC199541.4_FGT004*; Table 2). Taken together, these results suggest that, as previously observed in cucumber leaves after 3 h of N starvation [12], also in maize roots the shortage of this nutrient could have a stimulatory impact on anthocyanin synthesis even in a longer time (4 d).

It has been proved in *Arabidopsis* that the regulation of lateral root formation based on the cross-talk of JA and auxin is mediated by an ERF protein (AtERF109) that controls the expression of *AtASA1* and *AtYUC2* encoding key enzymes for auxin biosynthesis [25]. The positive modulation of a transcript (*GRMZM2G017193_T01*, Table 2) encoding a protein homologous to the flavin monooxygenase-like enzyme AtYUC2 suggests that auxin could play a role in lateral root formation in response to growth without N using a similar way of integrating JA- and auxin signaling. Anyway, the downregulation of two transcript for a class III peroxidase and a pectin lyase-like protein, respectively (Table 2) allows for hypothesizing that the N shortage negatively regulates the molecular mechanisms playing a role in cell wall loosening at the level of root elongation zone [26,27].

### 2.2. Root Transcriptional Responses Specific for the High-NUE Line Lo5

**Figure 2:**
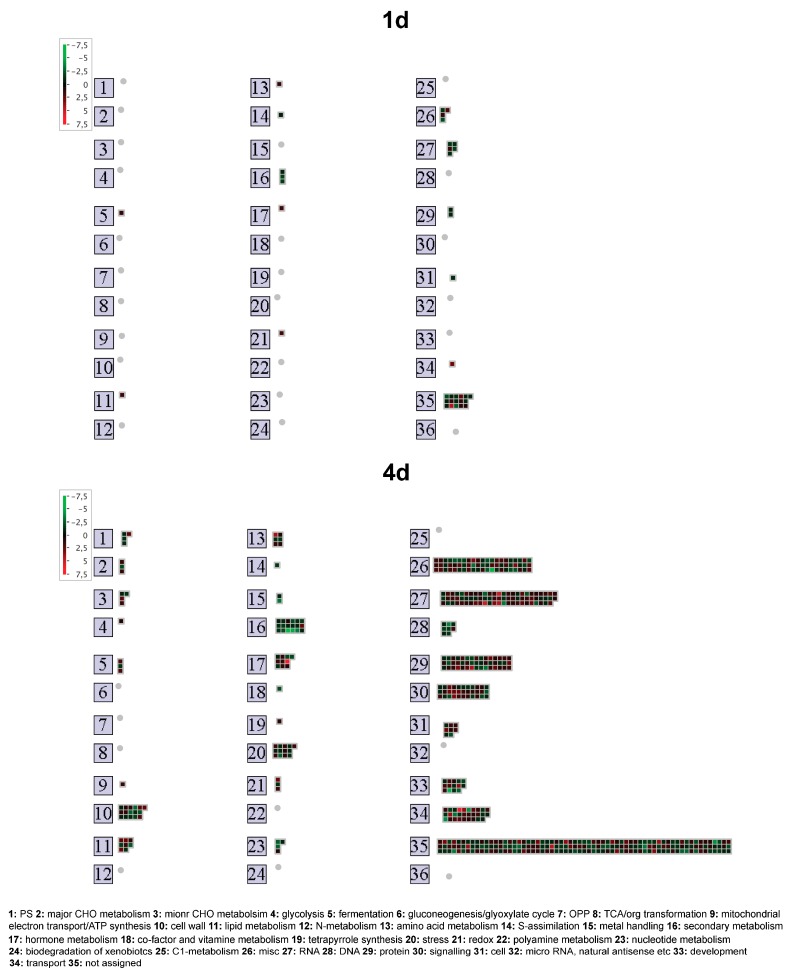
Overview of transcripts specifically modulated in Lo5 roots at 1 d and 4 d according to MapMan bincode classification. Log_2_(ratio) is shown by the color scale (green indicates a decrease and red an increase in transcript abundance in the comparisons 1 d vs. 0 d and 4 d vs. 0 d). Colored square: modulate transcript; gray circle: category without modulated transcripts.

Concerning the root transcriptional responses specific for Lo5 during the growth without N it turned out that 38 and 555 transcripts were modulated only in this inbred line after 1 d and 4 d respectively (Figure 1A,B, Table S3). MapMan overview of the distribution of these genes in main functional categories (Figure 2) underlined that at 1 d N shortage the most affected transcripts are not assigned to a specific metabolic pathway (Figure 2). The other most modulated categories are RNA metabolic processes, secondary metabolism and protein metabolic processes. All of them, in general, in a negative way (Figure 2). Some positively modulated transcripts are involved in metabolic pathways such as lipid metabolism, metabolism and transport of amino acid, hormone metabolism and redox processes (Figure 2). Also, after 4 d of N deprivation, the higher number of modulated genes are not assigned to a specific pathway or process and RNA metabolic processes (Figure 2). However, at this time we observed a higher number of transcripts involved in RNA metabolic process (e.g., regulation of transcriptions) that are positively affected by N deprivation. A similar trend can be observed for transcripts linked to protein metabolic processes (e.g., protein degradation and post-translational modification) (Figure 2). Other abundant categories are represented by signaling and transport processes (Figure 2). Like at 1 d, we observed a predominant upregulation of amino acid and lipid metabolic pathways, hormone metabolism and redox processes (Figure 2). In addition, at this time N shortage affected in Lo5 roots the expression of transcripts involved in cell wall metabolism and in the responses to stresses (Figure 2).

Delving deeper into the analysis of these specific changes in gene expression both after 1 and 4 d in Lo5 inbred line, N shortage had an impact on the metabolism of secondary compounds, in particular phenolics, and on cell wall metabolism (Table S3). In particular, focusing on the synthesis of amino acid precursor of phenylpropanoids, the 4-d N-shortage caused a different impairment on the synthesis on the phenylalanine (Phe) and tyrosine (Tyr) biosynthetic pathways as showed by the downregulation of a transcript for a chorismate mutase (CM, *AC198937.4_FGT003*) and an upregulation of another one encoding arogenate dehydratase (PDT, *GRMZM2G437912_T01*) (Table S3) suggesting specific positive effects on the synthesis of Phe [28]. Concerning phenylpropanoid biosynthesis, we observed a reduction of the expression level of a chalcone synthase (CHS) transcript after 4 d (*AC191551.3_FGT003*) with a concomitant increase for a chalcone-flavanone isomerase (CHI, *GRMZM5G882986_T01*) transcript (Table S3). Based on these results it can be inferred a positive modulation in Lo5 roots of the synthesis of flavanone-based flavonoids among which anthocyanins supported by the upregulation of a ferulic acid 5-hydroxylase (FAH, *AC210173.4_FGT005*, Table S3). In fact, it was shown that *AtFAH1* plays a role in anthocyanin biosynthesis under stress condition in *Arabidopsis* [29,30]. Other class of secondary metabolites resulted affected by the N deficiency. A negative regulation of terpenoid metabolic processes linked to gibberellin biosynthesis was recorded in particular at 4 d as proved by the repression of transcripts encoding a geranylgeranyl pyrophosphate synthase and two terpenoid cyclases/protein prenyltransferases (ent-kaurene synthases, KS) [31], respectively (Table S3). The hypothesized reduction of gibberellins (GA) in roots of Lo5 in response to N shortage could be involved in the control of root growth. It was reported that GA can control the root growth [27,32] through the modulation of the expression of xyloglucan endotransglycosylase/hydrolase (XTH) genes playing a role in cell wall loosening in the elongation zone [27]. Even though no modulation of (XTH) transcripts was recorded in Lo5 at 4 d, we observed a general downregulation of those encoding expansins (EXP, Table S3) that take part in the same process [27]. Besides, the induction of a transcript encoding a cellulase (CEL) and the repression of another one for cellulose synthase (CESA) suggest that secondary cell wall deposition can be positively affected (Table S3) [27]. However, another transcript encoding a protein showing homology to the *Arabidopsis* cellulose synthase-like D 3, AtCLSD3 (*GRMZM2G367267_T01*, Table S3) was repressed. AtCLSD3 plays a role in root growth and cell elongation in response to phosphate (Pi) starvation [33] suggesting an involvement of different cellulase genes on root morphology in a nutrient-specific way. Taken together, these results reinforce the hypothesis that the N deprivation could cause a negative impairment of molecular mechanism linked to the root elongation zone whilst positive effects on processes that occurs in the root differentiation zone. In addition, it was reported that GAs negatively control root growth and the increase of NO_3_^−^ uptake in cucumber not only under conditions of short-term suboptimal root-zone temperatures but also under optimal temperature condition [34]. In this context, the hypothesized reduction of GA in Lo5 roots could be linked to the repression of the *ZmNRT2.2* (*GRMZM2G010251_T01*, Table S3) after 4 d of growth without N. Moreover, at the same time point we recorded an upregulation of *ZmAMT3.3* (*GRMZM2G043193_T01*, Table S3). Conversely, Dechorgnat et al. [35] observed in the maize inbred B73 an opposite transcriptional behavior for *ZmNRT2.2* in plants growth under sufficient and low N conditions. Furthermore, in the same experiment the expression of *ZmAMT3.3* expression was independent of N levels. Based on our results, it is possible to infer that the expression of genes involved in NO_3_^−^ and NH_4_^+^ uptake can be genotype-specific or dependent on the experimental conditions.

Among the differentially expressed transcripts identified in Lo5 roots, some of them play a role in the transport of other nutrients, such as phosphorous (P) and magnesium (Mg) (Figure 2 and Table S3). In particular, N starvation induced three transcripts encoding high affinity P_i_ transporter, ZmPHT1;2 (*GRMZM2G139639_T01*), ZmPHT1;6 (*GRMZM5G881088_T01*) and ZmPHT1;8 (*GRMZM2G045473_T01*) [36] and the transcript for the Mg transporter ZmMGT3 (*GRMZM2G064467_T01*) [37] (Table S3). These results suggest that the growth without N can positively affect the uptake of other macronutrients such as P and Mg. However, in the case of sulfur (S), after 1 d and 4 d we recorded a negative modulation of a transcript for an adenosine 5′-phosphosulfate (APS) reductase (APR, *AC189750.4_FGT004*). This result agrees with the previously reported negative impact on gene expression and activity concerning APRs in *Arabidopsis thaliana* roots under N starvation [38].

Different actors can play a role in the signal transduction at the basis of these Lo5-specific root responses such as kinases. Interestingly, an increase in expression levels of a transcript (*GRMZM2G344388_T01*, Table S3) encoding a protein showing homology to AtMKK7 belonging to the MKK7-MKK6 module, a suppressor of meristem activity [39], can be linked to the hypothesized reduction of root growth in Lo5 under N starvation. Besides, the upregulation of the homologous to the *Arabidopsis* mitogen-activated protein kinase phosphatase PP2C5 (*GRMZM2G077187_T01*, Table S3) reinforces this hypothesis. AtPP2C5 negatively regulates activation of stress-induced MPK3, MPK4, and MPK6 [40]. Conversely, we also recorded a downregulation of transcript (*GRMZM2G172081_T01*, Table S3) encoding a protein showing homology to the plasma membrane-associated proline-rich extensin-like receptor kinase 4 (AtPERK4) that negatively regulates the root growth in the early stages of ABA signaling affecting the Ca^2+^ homeostasis [41]. Ca^2+^ has been hypothesized playing a role in NO_3_^-^ signaling as second messenger [42] but it could act also in the response to N starvation. In fact, a main upregulation of transcripts for calmodulin, calmodulin binding protein and calcium-binding EF-hand protein transcripts was detected in Lo5 roots after 4 d (Table S3). Furthermore, Lo5 roots appear to respond to N starvation through an alteration of cellular compartment Ca^2+^ concentration, which was proved by the decreased expression level of a lower affinity cation/H^+^ exchanger transcript (CAX, *GRMZM2G011592_T01*, Table S3) [43]. This transcript encodes a protein showing homology to AtCAX1 that is involved in vacuolar storage of Ca^2+^ in mesophyll cells of *Arabidopsis* leaf controlling different processes such as cell wall extensibility as suggested by the altered expression of genes for enzymes involved in cell wall metabolism detected in the loss-of-function mutant *cax1/cax3* [44]. On the basis of data, in Lo5 roots the downregulation of this CAX transcript could be involved in the regulation of cell wall metabolism reducing the expression of the EXP, CESA, CEL and CLS genes.

Focusing on transcription factors with a role in the response to NO_3_^−^ signaling pathway [45], we observed the downregulation of two transcripts encoding an LOB domain-containing protein 37 (LBD37, *GRMZM2G017319_T01*) and a TEOSINTE BRANCHED1/CYCLOIDEA/PROLIFERATING CELL FACTOR (TCP, *AC234521.1_FGT006*), respectively (Table S3). LBD37, LBD38 and LBD39 are negative regulators of anthocyanin biosynthesis and N-response genes [46] justifying the hypothesized increase in the synthesis of these secondary metabolites and the recorded reduction of the expression of *ZmNRT2.2* in Lo5 roots. A downregulation of LBD37 gene in response to N deficiency was also detected among the early N-deprivation responses in *Arabidopsis* roots [47]. In this species, TCP20 factor is a part of NO_3_^−^ signaling under N starvation, modulating the growth of lateral roots [48] and expression of key genes which belong to the anion signaling and assimilation pathways except for *AtNRT2.1* and *AtNiR* (nitrite reductase) [49]. Our results underlined that Lo5 roots respond to the growth without N specifically affecting some component involved in N sensing but also in anthocyanins biosynthesis and in their morphology.

### 2.3. Root Transcriptional Responses Specific for the Low-NUE Line T250

**Figure 3:**
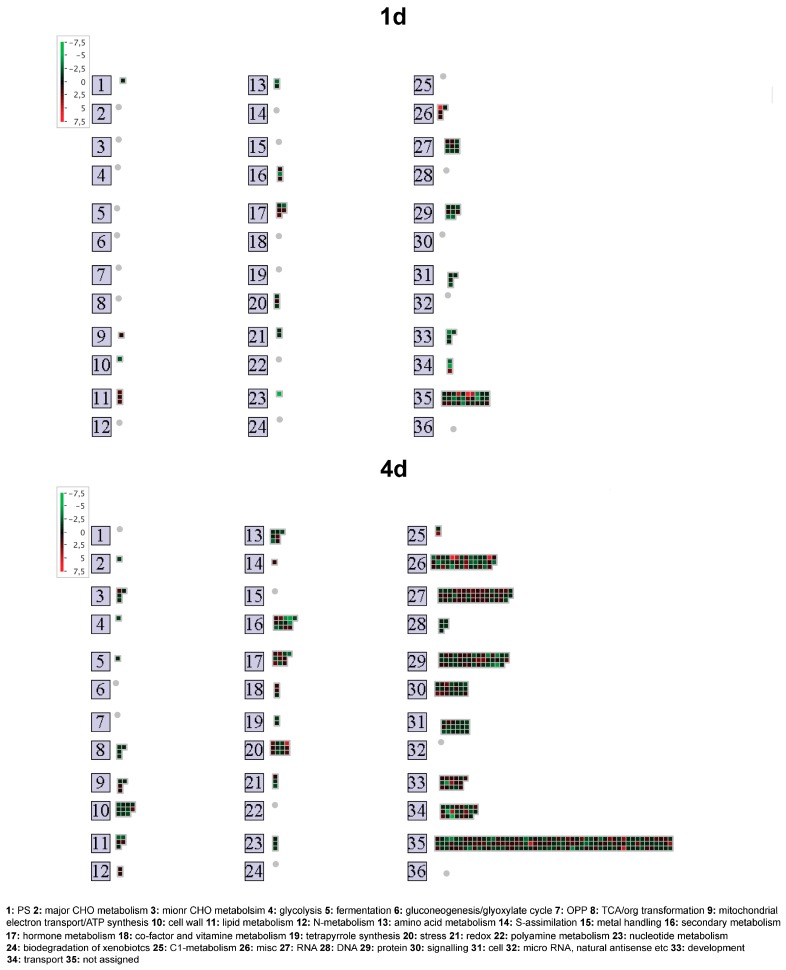
Overview of transcripts specifically modulated in T250 roots at 1 d and 4 d according to MapMan bincode classification. Log_2_(ratio) is shown by the color scale (green indicates a decrease and red an increase in transcript abundance in the comparisons 1 d vs. 0 d and 4 d vs. 0 d). Colored square: modulate transcript; gray circle: category without modulated transcripts.

T250 lines specifically affected 82 and 446 transcripts after 1 d and 4 d of N starvation, respectively (Figure 1). The overview of distribution in the main functional categories underlined a main reduction of expression of transcripts and showed that most of them are not assigned to a specific metabolic pathway both at 1 d and 4 d as for Lo5 line (Figure 3). Focusing on 1 d, we observed a general repression for transcripts related to protein and RNA metabolism, cell metabolism and development whilst hormone and lipid metabolisms were positively modulated (Figure 3). After 4 d of N deficiency, the main modulated functional categories are again protein and RNA metabolism and miscellaneous enzyme families, signaling, cell metabolism, development and transport (Figure 3). A negative impact of expression was observed in particular regarding cell wall metabolism, secondary compounds, amino acids and DNA (Figure 3).

Analyzing the T250-specific root transcriptional responses, we can hypothesize that also this maize inbred line responds to the growth without N affecting the metabolism of secondary compounds and cell wall. Focusing on phenylpropanoids, a main decrease of expression of genes involved in lignin branch pathway is underlined by the repression of transcripts encoding key enzymes of monolignol synthesis such as cinnamoyl-CoA reductase (CCR, *GRMZM2G017285_T01* and *GRMZM2G146031_T01*), cinnamyl alcohol dehydrogenase (CAD, *GRMZM2G046070_T01*) and hydroxycinnamoyl-coenzyme A shikimate/quinate hydroxycinnamoyltransferase (HCT, *GRMZM2G178769_T01*) (Table S3) [50,51]. Only one transcript for another HCT resulted upregulated at the same time point (*GRMZM2G089698_T01*; Table S3). Taken together, these results suggest that N shortage caused a negative impact on the lignification of cell wall linked to other specific modification of this cell compartment. In fact, N starvation had a negative impact on the levels of transcripts for pectin lyase (PL, *GRMZM2G179444_T01*), pectin methylesterase (PME, *GRMZM2G138999_T01*), CESA (*GRMZM2G082580_T01* and *GRMZM2G142685_T01*), XTH (*GRMZM2G388684_T01*) and EXP (*GRMZM2G094990_T01*) (Table S3) reinforcing the hypothesis of a block of the root growth under N starvation in maize roots [27]. In the case of T250 roots, we observed not only the downregulation at 1 d of a KS transcript involved in GA synthesis [31] (Table S3) but also of one encoding a XTH that can be induced by GA as previously mentioned [27]. Moreover, after 4 d peroxidase (PRX) genes were all repressed (Table S3) and this transcriptional behavior agreed with their hypothesized active role in cell wall loosening linked to root growth [27]. Moving to effects of N deficiency on nutrient transport and assimilation, contrarily to Lo5, in T250 roots was observed an increased expression of the *ZmNRT1.5* (*GRMZM2G044851_T01*, Table S3) at both time points, a reduction of *ZmPHT1;13* (*GRMZM2G070087_T01*) level and an increase in the expression of a APS kinase transcript (*GRMZM5G845021_T01*) at 4 d (Table S3). Interestingly, regarding the assimilatory pathway of N, we recorded the upregulation of the transcript encoding the plastidial glutamine synthetase 2 (ZmGS2) previously identified and characterized in roots of the same inbred line in response to the absence and/or different source of N [52]. As shown for Lo5, Mg transport also seems to be affected as suggested by the upregulation of a transcript encoding a Mg transporter (*ZmMGT7*, *GRMZM2G458879_T01*) (Table S3).

Concerning mechanisms linked to signal transduction in T250 roots under N shortage, we recorded the modulation of several transcripts encoding kinases and receptor-like kinase (Table S3) at the second time point. A gene encoding a PERK (*AC211175.3_FGT005*) showing homology to AtPERPK8 was repressed after 4 d (Table S3). AtPERPK8 was reported to be a negative regulator of root growth [53]. On the other hand, unlike Lo5, the regulation of cytosolic Ca^2+^ concentration seemed to be not affected in T250 since we did not detect the modulation of any CAX transcript. Only the repression of a gene encoding a protein homologous to the Ca^2+^-ATPase AtACA11 (*GRMZM2G476000_T01*, Table S3) was actually recorded. It was reported that this gene is not directly involved in the modulation of vacuolar Ca^2+^ concentration under stress condition as hypoxia [54]. On the other hand, other elements of Ca^2+^ homeostasis are influenced by N deprivation at 4 d in T250. In fact, in this inbred line an upregulation of a transcript encoding a transcription factor belonging to the family of calmodulin-binding transcription activators (CAMTAs) (*GRMZM2G447551_T01*, Table S3) was observed. CAMTAs are involved in responses to biotic and abiotic stresses in Arabidopsis and tomato [55].

As previously analyzed for Lo5, considering genes encoding transcription factors involved in NO_3_^-^ signaling pathway, in the low NUE line T250 we recorded after 4 d a downregulation of a transcript for the ortholog of AtLBD38 (*GRMZM2G177110_T01*), that with AtLBD37 and AtLBD39 plays a role the repression of N-responsive genes and genes involved in NO_3_^−^ assimilation [46]. Interestingly, the two maize inbred lines respond to growth without N affecting two different LBD proteins. Unlike *Arabidopsis*, where N deficiency simultaneously downregulated both *AtLBD37* and *AtLBD38* [47], in maize we observed a genotype-specific modulation of their two homologous genes. Conversely to Lo5, a positive modulation a transcript encoding a TCP factor (*GRMZM2G120151_T01*) at the same time point is recorded (Table S3). It could be hypothesized that in T250 roots the N starvation activates the NO_3_^−^-responsive genes as previously assumed in *Arabidopsis* where this activation is based on positive effects due to the interaction between NLP7 and TCP20 and negative regulation from LBD37/38/3 [45]. Only in T250 we found upregulation of genes involved in NO_3_^−^ transport (*ZmNRT1.5*) and N assimilation (*ZmGS2*) whilst the *ZmNRT2.2* was repressed in Lo5 roots.

### 2.4. Time Course of NO_3_^−^ and NH_4_^+^ Uptake Rates during the Growth in N Starvation in the Two Inbred Lines

**Figure 4:**
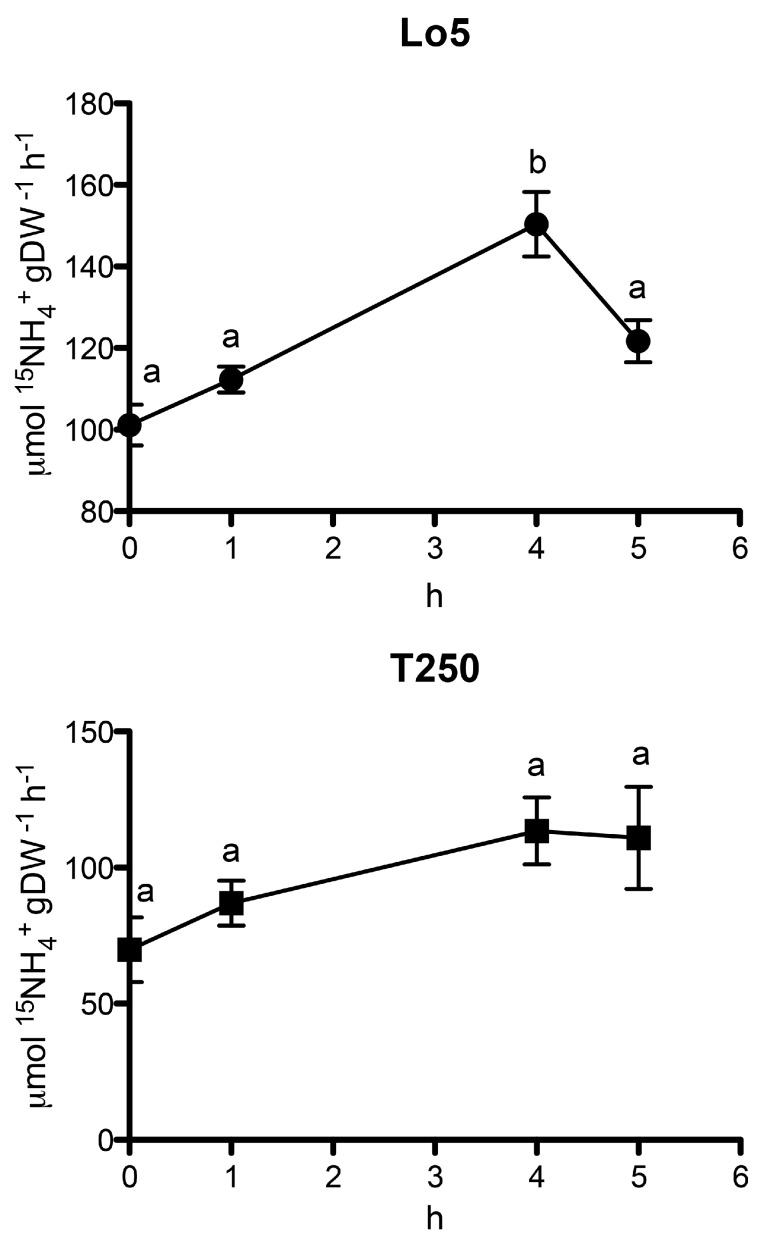
^15^NH_4_^+^ uptake rate during the growth in N-starvation (100 μM ^15^NH_4_^+^; mean ± S.E. of three biological replicates). ANOVA test was performed using GraphPad Prism^®^ (*n* = 3, *p* < 0.01; Tukey method, 95% confidence interval).

**Figure 5:**
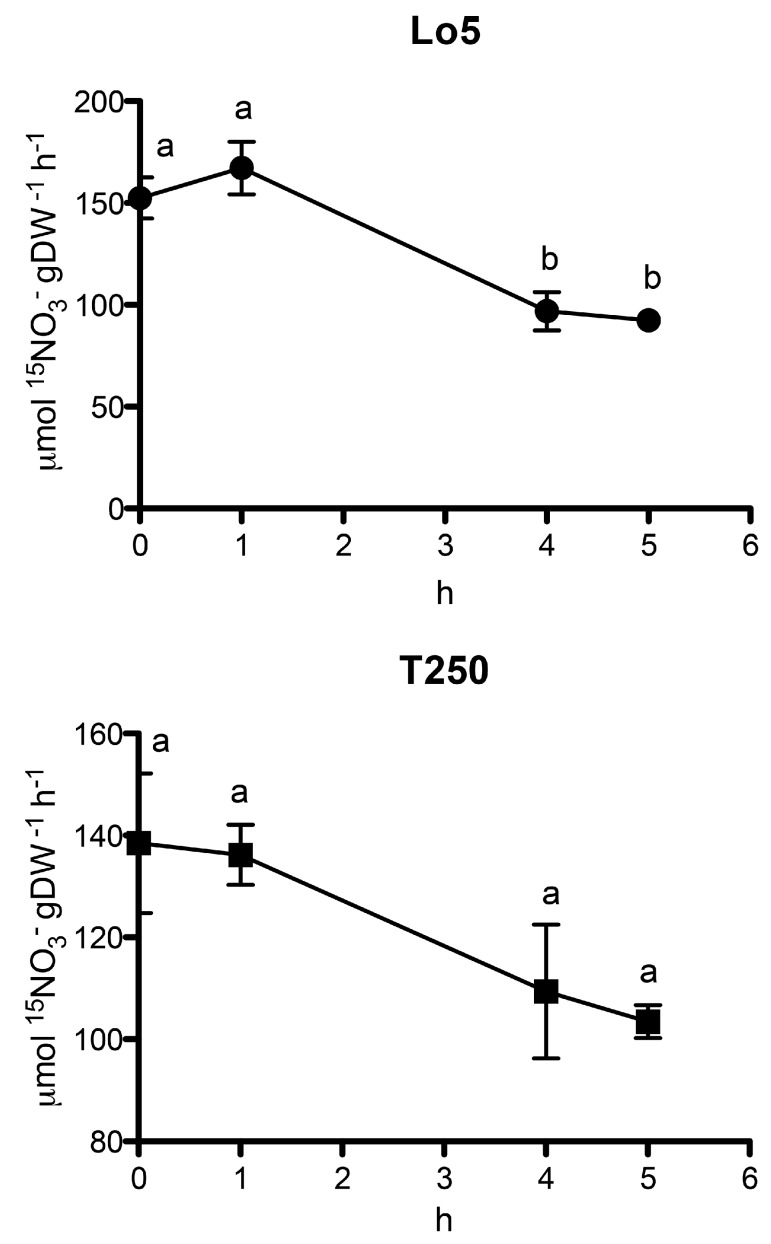
^15^NO_3_^−^ uptake rate during the growth in N-starvation (200 μM ^15^NO_3_^−^; mean ± S.E. of three biological replicates). ANOVA test was performed using GraphPad Prism^®^ (*n* = 3, *p* < 0.01; Tukey method, 95% confidence interval).

The transcriptional differences between Lo5 and T250 observed for transcripts involved in NO_3_^−^ and NH_4_^+^ transport during the growth without N are in line with the rate of ^15^NH_4_^+^ and ^15^NO_3_^−^ uptake determined at 0, 1, 4 and 5 d for both inbred lines (Figure 4 and Figure 5). The growth without N positively and negatively affected the uptakes rates of NH_4_^+^ and NO_3_^−^, respectively. Anyway, the differences are statistically significant only for the high NUE line, Lo5. In particular, in this line, the uptake rate of the cation peaked at 4 days of N starvation (Figure 4). This behavior agrees with the upregulation of the transcript for a high affinity NH_4_^+^ transporter, *ZmAMT3.3*, that was observed at 4 d only in this inbred line thus suggesting its possible involvement in the uptake process (Tables S1 and S3). NH_4_^+^ uptake rate trend is in line with previously results reported in roots of maize plants subjected to N starvation even though accompanied by a reduction of the expression of *ZmAMT1.1a* and *ZmAMT1.3*, the only two genes whose expression was analyzed [56]. A recent transcriptional analysis carried out in B73 maize roots [35] indicated the *ZmAMT1.1A*, *ZmAMT2.1* and *ZmAMT3.2* as genes modulated by N starvation. The results here obtained on the expression of the NH_4_^+^ transporter genes—that are also supported by the changes in NH_4_^+^ uptake rate—suggest that the effect of N starvation on NH_4_^+^ transport phenomena is strongly dependent on the genotypes and/or the experimental conditions.

NO_3_^−^ uptake rate shows a significant decrease after 4 d of growth without N only in Lo5. This behavior agrees with the downregulation of *ZmNRT2.2* observed at the same time point only in this line (Tables S1 and S3). The role of *ZmNRT2.2* in the response to low NO_3_^−^ in a genotypic-specific way was recently described [6]. According to this, a correlation between its changes in expression between low and adequate NO_3_^−^ concentration and the genotype-specific capability to maintain biomass under low N condition was shown [6]. Furthermore, an increase of *ZmNRT2.2* in maize roots under N starvation relative to the control was reported by Dechorgnat et al. [35]. Conversely, in our experiment we observed a decrease of the anion uptake rate (Figure 5) paralleling the *ZmNRT2.2* expression in the high NUE line. Again, these differences could be ascribed to the experimental conditions and genotype-based characteristic [6,35].

### 2.5. Conclusion

**Figure 6:**
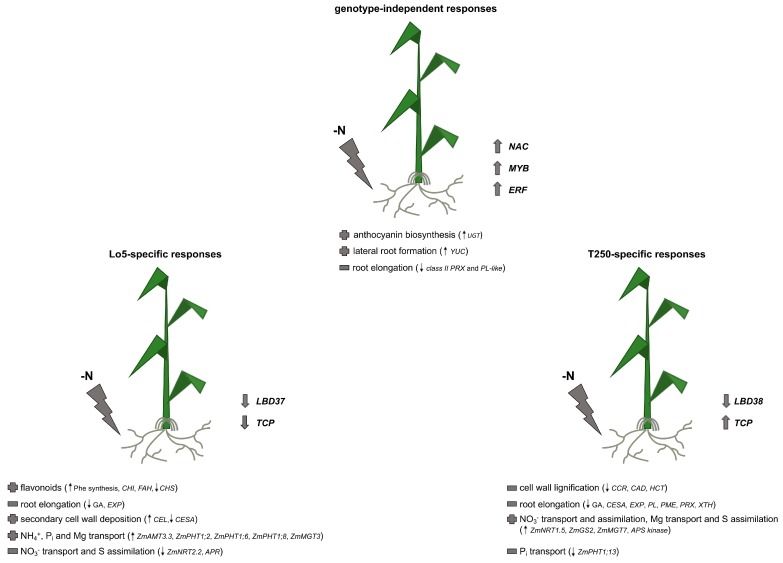
Schematic representation of genotype-independent and Lo5- and T250-specific root responses to the growth without N. Abbreviations: AMT1, AMMONIUM TRANSPORTER/METHYLAMMONIUM PERMEASE/RHESUS (AMT/MEP/RH) 1; APS KINASE, ADENOSINE 5′-PHOSPHOSULFATE KINASE; APR, ADENOSINE 5′-PHOSPHOSULFATE REDUCTASE; CAD, CINNAMYL ALCOHOL DEHYDROGENASE; CCR, CINNAMOYL-CoA REDUCTASE; CEL, CELLULASE; CESA, CELLULOSE SYNTHASE; CHI, CHALCONE-FLAVONE ISOMERASE; CHS, CHALCONE SYNTHASE; ERF, ETHYLENE RESPONSIVE ELEMENT BINDING FACTOR; EXP, EXPANSIN; FAH, FERULIC ACID 5-HYDROXYLASE; GA, GIBBERELLIN; GS2, GLUTAMINE SYNTHETASE; HCT, HYDROXYCINNAMOYL-COENZYME A SHIKIMATE/QUINATE HYDROXYCINNAMOYLTRANSFERASE; LDB, LOB DOMAIN-CONTAINING PROTEIN; NAC, NO APICAL MERISTEM (NAM), ARABIDOPSIS TRANSCRIPTION ACTIVATION FACTOR (ATAF), CUP-SHAPED COTYLEDON (CUC); MGT, MAGNESIUM TRANSPORTER; NRT1, NITRATE TRANSPORTER 1; NRT2, NITRATE TRANSPORTER 2; Phe, phenylalanine; PHT1, PHOSPHATE TRANSPORTER 1; PL, PECTATE LYASE; PL-like, PECTIN LYASE-like; PME, PECTIN METHYLESTERASE; PRX, PEROXIDASE; TCP, TEOSINTE BRANCHED1/CYCLOIDEA/PROLIFERATING CELL FACTOR; XTH, XYLOGLUCAN ENDOTRANSGLYCOSYLASE/HYDROLASE; UGT, UDP-GLUCOSYL TRANSFERASE; YUC, FLAVIN MONOOXYGENASE-LIKE. Top arrow: positive modulation; down arrow: negative modulation.

In Figure 6 we depicted the main putative genotype-independent and Lo5- and T250-specific root responses to growth without N. Even if different sets of transcripts are involved in the two lines, some processes such as the positive modulation of anthocyanin biosynthesis and lateral root formation and the negative impact on root elongation seem to be a general response to N deficiency in maize roots. On the other hand, we found strong differences in the ability to modulate the transcripts involved in the transport, distribution and assimilation of mineral nutrients (e.g., N, P and Mg). Although it is not possible to draw a definitive picture of key elements linked to the NUE, our data underlines the importance of the ability to differentially utilize N mineral forms. Only the Lo5 line, more efficient in field under low-N fertilization conditions (Rizzi), is able to respond to N starvation by increasing the NH_4_^+^ fluxes. It is conceivable that this form also absorbed on soil negatively charged particles is less subject to fluctuation and displaceable by root activity during the growth in a low N condition.

## 3. Material and Methods

### 3.1. Plant Material

Seeds of two maize inbred lines with different NUE (Lo5, high NUE and T250, low NUE), previously soaked in running water for 24 h, were allowed to germinate in the dark in a growth chamber at 26 °C for 4 d. The seedlings were then transferred in pots (12 plants for each pot) containing 2.2 L of 0.5 mM CaSO_4_ aerated solution for 48 h in controlled climatic conditions (day/night photoperiod, 16 h/8 h; Photosynthetically Active Radiation, PAR, 220 µE m^−2^s^−1^; temperature 27 °C). After this period, seedlings were subsequently grown for other 4 d using a nutrient solution (NS) without N with the following composition: 100 µM MgSO_4_, 200 µM K_2_SO_4_, 400 µM CaSO_4_, 175 µM KH_2_PO_4_, 5 µM KCl, 0.05 µM NaMoO_4_, 2.5 µM H_3_BO_3_, 0.2 µM MnSO_4_, 0.2 µM ZnSO_4_, 0.05 µM CuSO_4_, and 2 µM Fe-EDTA, pH = 6.0. The nutrient solution was replaced every 4 d. The root tissues of three seedlings were pooled after 0, 1 and 4 d of growth and used to extract total RNA for microarray and Real-time RT-PCR analyses. For each line (Lo5 and T250), six seedlings (technical replicates) were sampled at 0, 1, 4 and 5 d in order to determine the NH_4_^+^ and NO_3_^−^ uptake rate for each sampling time point (three seedlings for NH_4_^+^ and three seedlings for NO_3_^−^). This growth experiment was repeated three independent times (biological replicates). For each growth experiment (biological replicate) at least 4 pots were set-up in order to produce plant materials necessary for the microarray analysis and determination of NH_4_^+^ and NO_3_^−^ uptake rates.

### 3.2. Determination of NH_4_^+^ and NO_3_^−^ Uptake Rates

For each sample and for each biological replicate (one growth experiment) three seedlings were used (technical replicates) to determine the NH_4_^+^ uptake rate and three seedlings (technical replicates) to determine NO_3_^−^ uptake rate, respectively. The assay was performed with the seedlings of Lo5 and T250 grown for 0, 1, 4 and 5 d without N. The assays were repeated for each independent growth experiment (biological replicates). The seedling roots were washed for 5 min in a 0.5 mmol/L CaSO_4_ solution before transferring them to in the aerated uptake solution for 5 min. The uptake experiments were carried out incubating the roots of each plant in 25 mL of the uptake solution at 26° C. The uptake solutions for NH_4_^+^ and NO_3_^−^ assays consisted in 50 μM (^15^NH_4_)_2_SO_4_ (98 atom% ^15^N) and 100 μM Ca(^15^NO_3_)_2_ (99 atom% ^15^N) respectively in 1 mM MES-BTP (pH 6.0). After the incubation in the uptake solution, roots were washed for 1 min in 25 mL of a solution 0.5 mM CaSO_4_ at 4° C. Roots were then cut and treated at 70 °C for 48 h and ground to powder. The total N and ^15^N contents of each sample were determined through isotope ratio mass spectrometry analysis coupled with an elemental analyzer (DeltaV IRMS, Thermos Scientific, Waltham, MA, USA).

### 3.3. Microarray Analysis

Total RNA was extracted from root samples using the Spectrum^TM^ Plant Total RNA kit (Sigma-Aldrich, St. Louis, MO, USA), quantified using NanoDrop^TM^ 1000 (Thermo Scientific, Waltham, MA, USA). The RNA quality was evaluated through Agilent 2100 Bioanalyzer (Agilent Technologies, Santa Clara, CA, USA). For each sample, the cRNA synthesis and labeling were performed with 200 ng of total RNA and using the Low Input Quick Amp Labeling Kit, One-Color (Agilent) and Cyanine 3 (Cy3)-CTP fluorescent dye following the instructions of the Agilent technical manual (http://www.agilent.com). Cy3- labeled cRNA (1.65 μg) of each sample was hybridized on one sub-array of the custom 4 × 44K Agilent array (GPL22578) [48] according to manufacturer’s manual for 17 h at 65 °C. Array hybridizations and washing were performed according to manufacturer’s manual (One-Color Microarray-Based Gene Expression Analysis—Low Input Quick Amp Labeling—Protocol). Each chip was scanned on Agilent G2565CA Microarray Scanner System (Agilent) according to the instructions of the Agilent technical manual (http://www.agilent.com). Feature intensities were extracted using Agilent’s Feature Extraction Software 10.5.1.1 (Agilent). The hybridization data were normalized using the value of the 75th percentile. Differentially expressed transcripts were identified carried out transcriptional profile comparisons by t-test analysis performed using MeV software (http://mev.tm4.org/#/welcome) setting the following parameters: equal variance, alpha (overall threshold *p*-value): 0.01 and *p*-value based on t-distribution. Differentially expressed transcripts were filtered on the basis of |Log_2_(ratio)|≥ 1. Data are available at the GEO (http://www.ncbi.nlm.nih.gov/geo) under the series entry (GSE135613). For each transcript was used the annotation for the B73 reference genome (ftp://ftp.gramene.org/pub/gramene/maizesequence.org/release-5b/).

### 3.4. Real-Time RT-PCR Analysis

A set of differentially expressed transcripts were also analyzed by Real-time RT-PCR analysis with the same RNA samples used in microarray analysis. Primers sequences are shown in Table S4. Two transcripts encoding a putative translation elongation factor Tu family protein isoform 1 (*GRMZM2G153541_T01*) and a polyubiquitin containing 7 ubiquitin monomers (*GRMZM2G118637_T01*) respectively were used to normalized data. DNase I treatment was carried out for each sample using one microgram of total RNA and the RQ1 RNase-Free DNase (Promega, Madison, WI, USA) according to the manufacturer’s procedure. The samples were then used to produce cDNA using the ImProm-II Reverse Transcription System (Promega). Real-time RT-PCR experiments were carried out with the FastSYBR^®^ Green Master Mix (ThermoFisher Scientific) using the StepOnePlus^TM^ (ThermoFisher Scientific) according to the manufacturer’s protocols. The reactions were performed with a final volume of 10 μL, the primer concentration of 350 nM and 1 μL of a 1:4 solution of cDNA with the following thermal profile: 95 °C for 20 s, 40 cycle of 95 °C for 3 s and 60 °C for 30 s. PCR reaction efficiencies were calculated with the LinRegPCR program [57]. Mean normalized expression (MNE) [58] was calculated for each transcript and each sample using separately the two housekeeping transcripts. A mean MNE value was determined using a geometric mean of the two MNE values obtained for each transcript and each sample [59]. These MNE values were then used to calculate fold change (FC) values between samples considered in each comparison taken in account.

### 3.5. MapMan Analysis

Differentially expressed transcripts were mapped to bincode overview using MapMan tool [60] (https://mapman.gabipd.org/) using the maize mapping file Zmays_181 (https://mapman.gabipd.org/mapmanstore).

## Figures and Tables

**Table 1:** Number of up- and downregulated transcripts identified in response to the growth without N for root tissues of Lo5 and T250 inbred lines (*p*-value ≤ 0.01 and |Log_2_(ratio)|≥ 1).

	Lo5		T250	
	1 d vs. 0 d	4 d vs. 0 d	1 d vs. 0 d	4 d vs. 0 d
upregulated	15	294	31	206
downregulated	24	297	52	276

**Table 2:** Transcripts commonly modulated between the two inbred lines at 1 and 4 d of growth without N.

**1 d**			
**Transcript ID**	**Description**	**Log_2_(1 d/0 d) Lo5**	**Log_2_(1 d/0 d) T250**
GRMZM5G841684_T01	no hits found	−3.35	−1.91
**4 d**			
**Transcript ID**	**Description**	**Log_2_(4 d/0 d) Lo5**	**Log_2_(4 d/0 d) T250**
GRMZM2G145458_T01	jasmonate-zim-domain protein 11	1.54	1.11
GRMZM2G173965_T01	mitogen-activated protein kinase kinase kinase 15	1.91	1.75
GRMZM2G565911_T01	Zinc finger (C3HC4-type RING finger) family protein	−2.05	−2.21
GRMZM2G043336_T01	no hits found	−2.69	−4.55
AC186789.4_FGT001	no hits found	−1.49	−2.32
GRMZM2G425638_T01	germin-like protein subfamily 2 member 2 precursor	−2.22	−1.88
GRMZM2G138450_T01	peroxidase superfamily protein	−9.51	−7.76
GRMZM2G080992_T01	MATE efflux family protein	1.72	1.25
GRMZM2G074401_T01	fatty acid desaturase 8	1.22	1.36
GRMZM2G318843_T01	calmodulin-binding family protein	−2.95	−1.43
GRMZM2G168747_T01	natural resistance-associated macrophage protein 1	2.89	4.62
GRMZM2G325462_T01	no hits found	2.58	2.89
GRMZM5G824574_T01	protein of unknown function (DUF569)	−1.93	−1.10
GRMZM2G347766_T01	nucleotide-diphospho-sugar transferases superfamily protein	2.17	2.16
GRMZM2G075461_T01	cytochrome P450, family 709, subfamily B, polypeptide 2	2.09	2.26
GRMZM2G162739_T01	NAC (No Apical Meristem) domain transcriptional regulator superfamily protein	1.75	1.62
GRMZM2G096269_T01	glutathione S-transferase phi 8	−1.08	−1.00
GRMZM2G134279_T01	myb domain protein 112	1.42	2.19
GRMZM2G028438_T01	SCARECROW-like 8	1.96	1.70
GRMZM2G379575_T01	no hits found	−2.01	−1.92
GRMZM2G024996_T01	no hits found	8.84	9.70
GRMZM2G044773_T01	RING/U-box superfamily protein	2.35	2.45
GRMZM5G876638_T01	no hits found	−1.80	−1.71
GRMZM2G455564_T01	pectin lyase-like superfamily protein	−1.41	−1.14
GRMZM5G807276_T01	2-oxoglutarate (2OG) and Fe(II)-dependent oxygenase superfamily protein	−3.80	−4.47
GRMZM2G420988_T01	ADP/ATP carrier 2	2.99	3.36
GRMZM2G120021_T01	basic helix-loop-helix (bHLH) DNA-binding superfamily protein	−1.56	−1.48
GRMZM2G080516_T01	ethylene responsive element binding factor 1	1.78	1.71
GRMZM2G017193_T01	flavin-binding monooxygenase family protein	2.24	2.23
GRMZM2G401606_T01	S-locus lectin protein kinase family protein	1.82	3.43
GRMZM2G178209_T01	UDP-glucosyl transferase 73D1	3.62	1.79
GRMZM2G127789_T01	glutathione S-transferase TAU 8	−2.05	−2.77
GRMZM2G110567_T01	zinc finger (C3HC4-type RING finger) family protein	−2.29	−2.66
GRMZM2G429035_T01	no hits found	−1.53	−1.22
GRMZM2G140101_T01	no hits found	−2.66	−3.93
AC199541.4_FGT004	UDP-glucosyltransferase 74F2	1.28	1.07

## Supplementary Materials

Supplementary materials can be found at https://www.mdpi.com/1422-0067/20/19/4856/s1.
