# Supplementary material for: Nitrogen Starvation Differentially Influences Transcriptional and Uptake Rate Profiles in Roots of Two Maize Inbred Lines with Different NUE
Source: Int J Mol Sci. 2019 Sep 30;20(19):4856. doi: 10.3390/ijms20194856 (PMC6801476; doi:10.3390/ijms20194856)
Supplement: Supplementary file 1 [file ijms-20-04856-s001.zip › ijms-595434 suppl for final/Supplementary files/Figure S1_Table S2_Table S4.pdf]

Figure S1

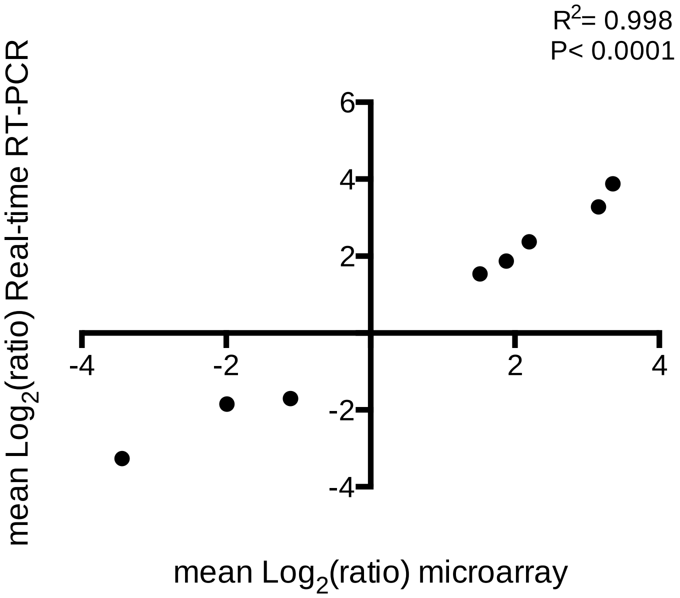

Correlation analysis of the mean Log_2(ratio) values obtained by Real-time RT-PCR and microarray analyses. Real-time RT-PCR validation were carried out for 8 differentially expressed transcripts identified in the 4 comparison of root transcriptional profile obtained through microarray analysis. The mean values were calculated using the values obtained for the three biological replicates of each sample. Pearson correlation analysis was carried out using a 95% confidence interval using the GraphPad Prism software (version 6.0).

Table S2

		Lo5 1d *vs* 0d		Lo5 4d *vs* 0d	
**Transcript ID**	**Description**	**FC microarray**	**FC Real-time RT-PCR**	**FC microarray**	**FC Real-time RT-PCR**
AC189750.4_FGT004	APS reductase 3	-2.16	-3.85 ± 0.94		
GRMZM2G010251_T01	ZmNRT2.2			-10.85	-11.36 ± 3.62
GRMZM2G011598_T01	NAC domain containing protein 47			3.68	3.66 ± 1.77
GRMZM2G036708_T01	cysteine synthase C1	2.88	2.91 ± 1.20		
GRMZM2G043193_T01	ammonium transporter 2, ZmAMT3.3			4.60	5.18 ± 1.20
		**T250 1d *vs* 0d**		**T250 4d *vs* 0d**	
**Transcript ID**	**Description**	**FC microarray**	**FC RT-PCR**	**FC microarray**	**FC RT-PCR**
GRMZM2G044851_T01	ZmNTR1.5	8.94	9.72 ± 1.47		
GRMZM2G044851_T01	ZmNTR1.5			10.27	14.70 ± 3.55
GRMZM2G156599_T01	YELLOW STRIPE like 3			-3.97	-4.19 ± 0.98

Results of Real-time RT-PCR analysis of a set of transcripts differentially expressed in Lo5 and T250 roots between different days of growth without N. Transcript ID, description, microarray fold change (FC) value and Real-time RT-PCR FC (means ± SE of three biological replicates) were reported.

Table S4

ProbeName	Transcript ID	Description	Forward primer	Reverse primer
CUST_38825_PI430257437	AC189750.4_FGT004	APS reductase 3	5'-CTCCAGTGAGCGAGAACCAT- 3'	5'-GTGTCACTACCAAACCCAGT- 3'
CUST_31215_PI430257437	GRMZM2G010251_T01	ZmNRT2.2	5'- ATGTTCACCTGCTACCTACC-3'	5'- GAATATCGTTGGCACATCTC-3'
CUST_21167_PI430257437	GRMZM2G011598_T01	NAC domain	5‘-ATGGGCACGATGAACTGACA-3‘	5‘-GTGTGTGTTTGGTGCAATAGAT-3‘
CUST_33358_PI430257437	GRMZM2G036708_T01	Cysteine synthase C1	5‘-CATTCCCCCCTCATCCAGTT-3‘	5‘-TCTCTCTTTCAACATTTTCCCAT-3‘
CUST_25268_PI430257437	GRMZM2G043193_T01	ZmAMT3.1	5'-GTGCATTTGTTTGCTAATTGCT-3‘	5‘-ATCCTGACACTCTACATTCCT-3‘
CUST_21617_PI430257437	GRMZM2G044851_T01	ZmNRT1.5	5'-GGCTTACGCATGACCATCA-3'	5'-CCTCCACCTCAAGAAGACAA-3'
CUST_22506_PI430257437	GRMZM2G156599_T01	YELLOW STRIPE like	5‘-GAGATACACTGAAGGCATCA-3‘	5‘-ACAAGGATAATACAGGGGACA-3‘

Sequence of forward and reverse primers used in Real-time RT-PCR experiments. ProbeName, Transcript ID, Description and 5'-3' sequences of forward and reverse primers were reported for each transcript.
