# Supplementary material for: Nitrogen Starvation Differentially Influences Transcriptional and Uptake Rate Profiles in Roots of Two Maize Inbred Lines with Different NUE
Source: Int J Mol Sci. 2019 Sep 30;20(19):4856. doi: 10.3390/ijms20194856 (PMC6801476; doi:10.3390/ijms20194856)
Supplement: Supplementary file 1 [file ijms-20-04856-s001.zip › ijms-595434 suppl for final/Supplementary files/suppl figure lengend and table caption.pdf]

Figure S1: Correlation analysis of the mean Log_2(ratio) values obtained by Real-time RT-PCR and microarray analyses.

Table S1: Differentially expressed transcripts identified by each comparison of transcriptional profiles (Lo5 1 d vs. od; Lo5 4 d vs. 0 d; T250 1 d vs. 0 d; T250 4 d vs. 0 d).

Table S2: Results of Real-time RT-PCR analysis of a set of transcripts differentially expressed in Lo5 and T250 roots between different days of growth without N.

Table S3: Transcripts specifically modulated in Lo5 and T250 lines at 1 and 4 d.

Table S4: Sequence of forward and reverse primers used in Real-time RT-PCR experiments.
